# Inactivation of the Host Lipin Gene Accelerates RNA Virus Replication through Viral Exploitation of the Expanded Endoplasmic Reticulum Membrane

**DOI:** 10.1371/journal.ppat.1003944

**Published:** 2014-02-20

**Authors:** Chingkai Chuang, Daniel Barajas, Jun Qin, Peter D. Nagy

**Affiliations:** Department of Plant Pathology, University of Kentucky, Lexington, Kentucky, United States of America; Institute of Microbiology at Chinese Academy of Science, China

## Abstract

RNA viruses take advantage of cellular resources, such as membranes and lipids, to assemble viral replicase complexes (VRCs) that drive viral replication. The host lipins (phosphatidate phosphatases) are particularly interesting because these proteins play key roles in cellular decisions about membrane biogenesis versus lipid storage. Therefore, we examined the relationship between host lipins and tombusviruses, based on yeast model host. We show that deletion of *PAH1* (phosphatidic acid phosphohydrolase), which is the single yeast homolog of the lipin gene family of phosphatidate phosphatases, whose inactivation is responsible for proliferation and expansion of the endoplasmic reticulum (ER) membrane, facilitates robust RNA virus replication in yeast. We document increased tombusvirus replicase activity in *pah1Δ* yeast due to the efficient assembly of VRCs. We show that the ER membranes generated in *pah1*Δ yeast is efficiently subverted by this RNA virus, thus emphasizing the connection between host lipins and RNA viruses. Thus, instead of utilizing the peroxisomal membranes as observed in wt yeast and plants, TBSV readily switches to the vastly expanded ER membranes in lipin-deficient cells to build VRCs and support increased level of viral replication. Over-expression of the *Arabidopsis* Pah2p in *Nicotiana benthamiana* decreased tombusvirus accumulation, validating that our findings are also relevant in a plant host. Over-expression of AtPah2p also inhibited the ER-based replication of another plant RNA virus, suggesting that the role of lipins in RNA virus replication might include several more eukaryotic viruses.

## Author Summary

Genetic diseases alter cellular pathways and they likely influence pathogen-host interactions as well. To test the relationship between a key cellular gene, whose mutation causes genetic diseases, and a pathogen, the authors have chosen the cellular lipins. Lipins are involved in a key cellular decision on using lipids for membrane biogenesis or for storage. Spontaneous mutations in the *LIPIN1* gene in mammals, which cause impaired lipin-1 function, contribute to common metabolic dysregulation and several major diseases, such as obesity, hyperinsulinemia, type 2 diabetes, fatty liver distrophy and hypertension. In this work, the authors tested if tomato bushy stunt virus (TBSV), which, similar to many (+)RNA viruses, depends on host membrane biogenesis, is affected by deletion of the single lipin gene (*PAH1*) in yeast model host. They show that *pah1Δ* yeast supports increased replication of TBSV. They demonstrate that TBSV takes advantage of the expanded ER membranes in lipin-deficient yeast to efficiently assemble viral replicase complexes. Their findings suggest possible positive effect of a genetic disease caused by mutation on the replication of an infectious agent.

## Introduction

Positive-stranded (+)RNA viruses are important and emerging human, animal and plant pathogens. These viruses utilize cellular membranes and lipids during replication to build viral replicase complexes (VRCs) [1]–[4]. The subverted subcellular membranes are proposed to provide critical lipid or protein cofactors to regulate the function of the viral replicase, serve as scaffolds for VRC assembly, provide protection of the viral RNA against cellular nucleases, prevent recognition by the host antiviral surveillance system, or facilitate the targeting of the viral replication proteins to a particular microdomain in the membrane [1]–[9]. (+)RNA viruses induce membrane proliferation that requires new lipid biosynthesis as shown by several genome-wide screens, which identified lipid biosynthesis/metabolism genes [10]–[15]. Accordingly, several examples of virus-induced modification of cellular lipid metabolism and changes in lipid composition of membranes during virus replication are documented in the scientific literature [16]–[20].

Tombusviruses, such as *tomato bushy stunt virus* (TBSV) (Table S1), are among the best-characterized viruses [3], [12], [13], [21]–[25]. They belong to supergroup 2 (+)RNA viruses that include animal flaviviruses, and pestiviruses and plant luteoviruses, carmoviruses, and others. Tombusviruses code for five proteins including two replication proteins, termed p33 and p92^pol^ [26]–[28]. Both p33 and p92^pol^ are translated from the genomic (g)RNA and p92^pol^ is the result of translational readthrough of the p33 stop codon [28], [29]. p92^pol^ is the RNA-dependent RNA polymerase [30]–[32], while p33 is an RNA chaperone playing a role in RNA template selection and recruitment and in the VRC assembly [32]–[37].

(+)RNA viruses either target existing subcellular membranes or they extensively remodel membranes to support the VRC assembly. Interestingly, tombusviruses can utilize pre-existing membranes, but also remodel subcellular membranes by forming multivesicular body-like structures in infected cells [5], [6], [38], [39]. Tombusviruses are useful for membrane remodeling studies, because they can utilize peroxisomal membranes [e.g., TBSV and the closely related *cucumber necrosis virus* (CNV)] [5], [39], or they can switch to endoplasmic reticulum (ER) membranes in the absence of peroxisomes [40], [41], or replicate in ER or mitochondrial membranes *in vitro* [42].

Genetic diseases can alter critical cellular processes, which might affect pathogens that have to take advantage of cellular resources. The host lipins are particularly interesting because these proteins play key roles in cellular decisions about membrane biogenesis versus lipid storage [43]–[45]. Spontaneous mutations in the *LIPIN1* gene in mammals, which cause impaired lipin-1 function, contribute to common metabolic dysregulation and several major diseases, such as obesity, hyperinsulinemia, type 2 diabetes, fatty liver distrophy and hypertension [44], [46], [47].

**Figure 1:**
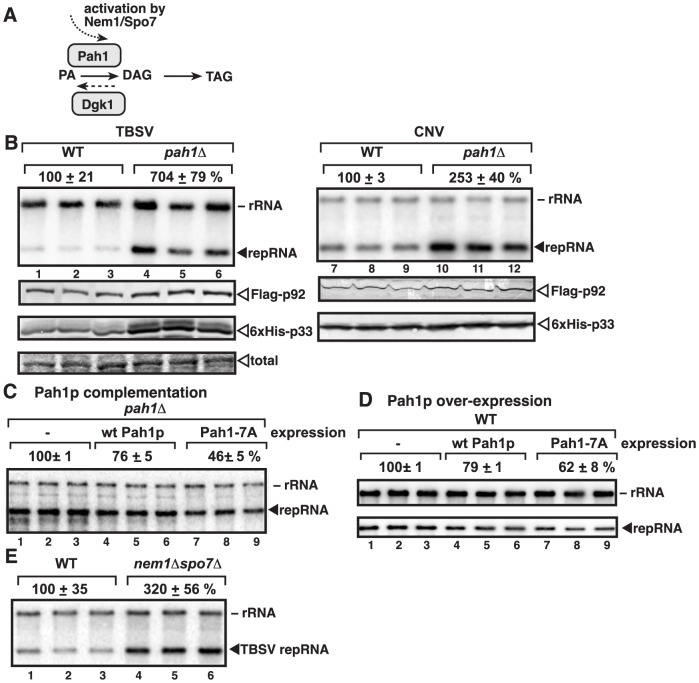
Deletion of the single yeast lipin gene (*PAH1*) enhances TBSV repRNA accumulation. (A) The role of Pah1p phosphatidate phosphatase and Dgk1p diacylglycerol kinase in lipid synthesis in yeast. To convert phosphatidic acid (PA) to diacylglycerol (DAG), Pah1p is dephosphorylated (activated) by the ER-localized Nem1p/Spo7p complex. (B) Top panel: Replication of the TBSV repRNA in wt and *pah1Δ* yeast was measured by Northern blotting 24 h after initiation of TBSV replication. Yeast co-expressed the TBSV (lanes 1–6) and the CNV (lanes 7–12) p33 and p92 replication proteins. The accumulation level of repRNA was normalized based on the ribosomal (r)RNA. Each sample is obtained from different yeast colonies. Middle and bottom panels: The accumulation levels of FLAG-p92 and 6×His-p33 were tested by Western blotting. Each experiment was repeated. (C–D) Expression of wt Pah1p and a phosphorylation deficient, constitutively active Pah1p, called Pah1-7A, which contains alanine substitutions for all seven phosphorylation sites, reduces TBSV replication in *pah1Δ* and wt yeasts. Northern blotting was done as in panel B. (E) Stimulatory effect of deletion of *NEM1* and *SPO7*, which form the dephosphorylation complex in the ER membrane, on TBSV repRNA accumulation is shown by Northern blotting. Note that Nem1p and Spo7p are required to dephosphorylate Pah1p, leading to the activation and relocalization of Pah1p from the cytosol to the ER membrane.

The yeast *PAH1* (phosphatidic acid phosphohydrolase) gene is the homolog of the mammalian fat-regulating protein Lipin-1 [43], [45], [48]. Like the three mammalian lipin genes, the single copy yeast *PAH1* codes for a phosphatidate phosphatase (PAP), which dephosphorylates phosphatidic acid (PA), yielding diacylglycerol (DAG) (Fig. 1A). Pah1p is involved in synthesis of DAG and triacylglycerol (TAG) storage lipids, and in the absence of *PAH1*, the ER/nuclear membrane expands considerably and the total phospholipid content of the cell increases by ∼2-fold [49], [50]. Thus, Pah1p sits at the crossroads between membrane biogenesis and lipid storage (i.e., the decision to store fat or build membranes) [45]. The mammalian or plant lipins can complement Pah1p function in yeast, demonstrating the functional similarity among these enzymes [46], [51]. Pah1p is the only yeast PAP protein involved in the synthesis of TAG and the regulation of phospholipid biosynthesis [52].

Since (+)RNA viruses likely depend on membrane biogenesis, we examined the relationship between the host lipins and tombusviruses, based on yeast model host. In this paper, we document that deletion of the yeast lipin gene, *PAH1*, whose inactivation is responsible for proliferation and enlargement of the ER membrane, facilitates robust RNA virus replication in yeast. Thus, surprisingly, a host gene whose homologs are involved in genetic diseases in humans, greatly affects virus replication.

## Results

### Deletion of yeast *PAH1* gene, a lipin ortholog, increases tombusvirus replication in yeast

Since TBSV and the closely related CNV (Table S1) induce membrane proliferation and they replicate by utilizing peroxisomal membranes for VRC assembly *in vivo* [5], [40]–[42] and ER membranes *in vitro* [42], we tested if deletion of *PAH1* gene, which leads to ER membrane enlargement and proliferation [43], [45], [46], could alter TBSV and CNV replication in yeast cells. We found that the TBSV replicon (rep)RNA accumulated to ∼7-fold higher in the presence of TBSV replication proteins (Fig. 1B, lanes 4–6 versus 1–3) and ∼2.5-fold higher levels in case of CNV (lanes 10–12 versus 7–9) in *pah1Δ* yeast. These data suggest that the enlarged ER might provide favorable microenvironment for TBSV and CNV replication or tombusviruses might be able to take advantage of the increased phospholipid content of the cell. Interestingly, the levels of p33 and p92^pol^ replication proteins were increased in *pah1Δ* yeast (Fig. 1B).

Moreover, expression of wt Pah1p protein in *pah1Δ* yeast had moderate inhibitory effect on TBSV replication (Fig. 1C, lanes 4–6) and over-expression Pah1p also inhibited TBSV replication (Fig. 1D, lanes 4–6). This moderate inhibitory effect by wt Pah1p could be due to phosphorylation and inactivation of the enzymatic function of the over-expressed Pah1p in yeast [50], [53]. Therefore, we also expressed/over-expressed a constitutively active, phosphorylation-deficient mutant of Pah1p, which indeed led to more pronounced inhibition of TBSV repRNA accumulation in *pah1Δ* or wt yeasts (by ∼40-to-50%, see mutant Pah1-7A containing alanine substitutions for all seven phosphorylation sites, lanes 7–9, Fig. 1C–D).

In addition, we tested TBSV accumulation in *nem1Δspo7Δ* yeast, which lacks the ER-associated phosphatase complex needed for ER association, dephosphorylation, and activation of Pah1p [50]. As expected, TBSV replication increased by ∼3-fold in *nem1Δspo7Δ* yeast (Fig. 1E, lanes 4–6 versus 1–3), further supporting the major role of Pah1p in TBSV replication.

Finally, we also tested the effect of over-expression of Dgk1p diacylglycerol kinase, which catalyzes the production of PA from DAG, the opposite reaction with Pah1p (Fig. 1A), on TBSV replication. Overproduction of the ER-localized Dgk1p induces the enlargement of ER-like membranes in yeast [54], [55]. We found that overproduction of Dgk1p in yeast led to increased TBSV replication (Fig. S1, lanes 4–6). Altogether, the above data support that the enlarged ER or the increased phospholipid content of the cell is a major advantage for TBSV and CNV replication.

**Figure 2:**
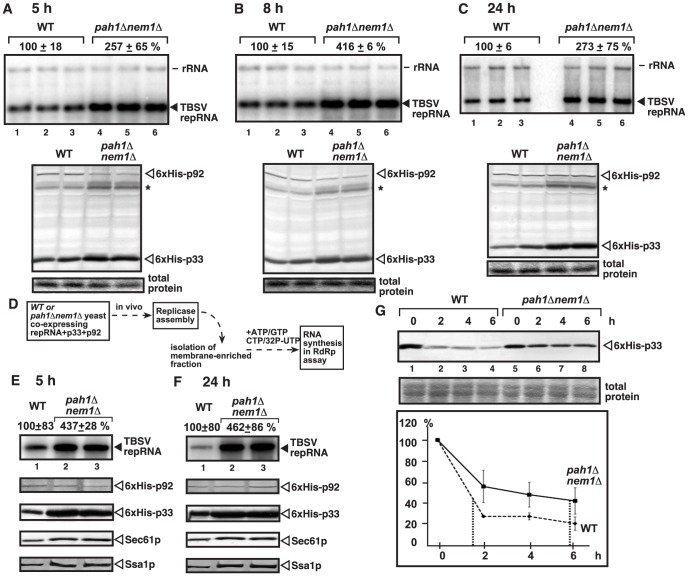
Increased TBSV repRNA replication and enhanced p33 stability in yeast lacking Pah1p. (A–C) Time points experiments to show the accumulation levels of the TBSV repRNA, 6×His-p33 (CNV) and 6×His-p92 (CNV) in wt and *pah1Δ nem1Δ* yeasts. Asterisk marks a detergent-resistant p33 dimer band. Note that this mutant yeast behaves similarly to *pah1Δ* yeast during tombusvirus replication. See details in Fig. 1B. (D) The scheme of the *in vitro* TBSV replication assay based on the isolated membrane fraction carrying the tombusvirus replicase and the bound RNA template. (E–F) Top panels: Increased *in vitro* replication of TBSV repRNA in the isolated membrane fraction from *pah1Δ nem1Δ* yeast when compared with that from wt yeast. Note that the levels of p92 replication protein were normalized as shown in the second panel. The third, fourth and fifth panels show the accumulation levels of 6×His-p33, and the cellular Sec61p (an ER marker) and Ssa1p Hsp70. Samples in panel E and F were taken at 5 and 24 h time points, respectively. (G) Increased stability of the p33 replication protein in yeast lacking Pah1p. The accumulation level of 6×His-p33 in wt and *pah1Δ nem1Δ* yeasts was measured by Western blotting at the shown time points. The production of 6×His-p33 (CNV) lasted for 3 h from the inducible *GAL1* promoter, followed by turning off transcription and stopping translation by changing the galactose media to a new media containing glucose and cyclohexamide (taken as “0 time point”). The p33 level at the 0 time point was taken as 100%.

To test if the high level of TBSV accumulation is due to increased TBSV repRNA replication, we measured the level of TBSV repRNA accumulation at various time points after induction of replication in *pah1Δnem1Δ* or *pah1Δ* yeast. These experiments revealed that TBSV repRNA accumulated to 2.5-to-4-fold higher level even at the early time points (Fig. 2A–B, 5 and 8 hour time points). Similarly, TBSV repRNA accumulation was ∼5-fold higher at an early time point in *pah1Δ* yeast (Fig. S2). These data suggest that more robust TBSV replication occurs earlier in *pah1Δnem1Δ* and *pah1Δ* yeasts than in the wt yeast, indicating that VRCs might be able to assemble faster in the mutant yeast cells.

Testing the replicase activity in the isolated membrane fraction containing the viral replicase/viral RNA complex from *pah1Δnem1Δ* (Fig. 2D–F) or *pah1Δ* yeast (Fig. S3A–B) also showed ∼4-to-5-fold increase over the replicase activity observed with the isolated membrane fraction from wt yeast at both early and late time points. The isolated membrane fractions were adjusted to contain comparable amount of the p92^pol^ replication protein (Fig. 2E–F, S3B), thus the increased *in vitro* replicase activity in the samples from *pah1Δnem1Δ* or *pah1Δ* yeasts indicates that the tombusvirus replicase in *pah1Δnem1Δ* or *pah1Δ* yeasts is more active than in wt yeast. Interestingly, unlike p92^pol^, the amounts of the tombusvirus p33 replication protein, the Sec61p ER resident protein and the Ssa1p Hsp70 chaperone, which is co-opted for TBSV replication, all increased in the isolated membrane fraction from *pah1Δnem1Δ* (Fig. 2D–E) or *pah1Δ* yeast (Fig. S3B). The presence of elevated amounts of p33 [56] and Ssa1p [37], [57], [58] has been shown to increase TBSV replication, which is in agreement with the increased *in vitro* replicase activity in the isolated membrane fraction from *pah1Δnem1Δ* or *pah1Δ* yeasts.

Since p33 replication protein is an integral membrane protein [39], [58], the observation of increased p33 level in the isolated membrane fraction from *pah1Δnem1Δ* yeast suggests that p33 might be more stable in the mutant yeast than in the wt yeast. Indeed, estimation of the half-life of p33 revealed ∼4-fold increased stability of p33 in *pah1Δnem1Δ* yeast in comparison with wt yeast (Fig. 2G).

### Enhanced *in vitro* assembly of the tombusvirus replicase complex in cell-free extract from yeast lacking *PAH1*

**Figure 3:**
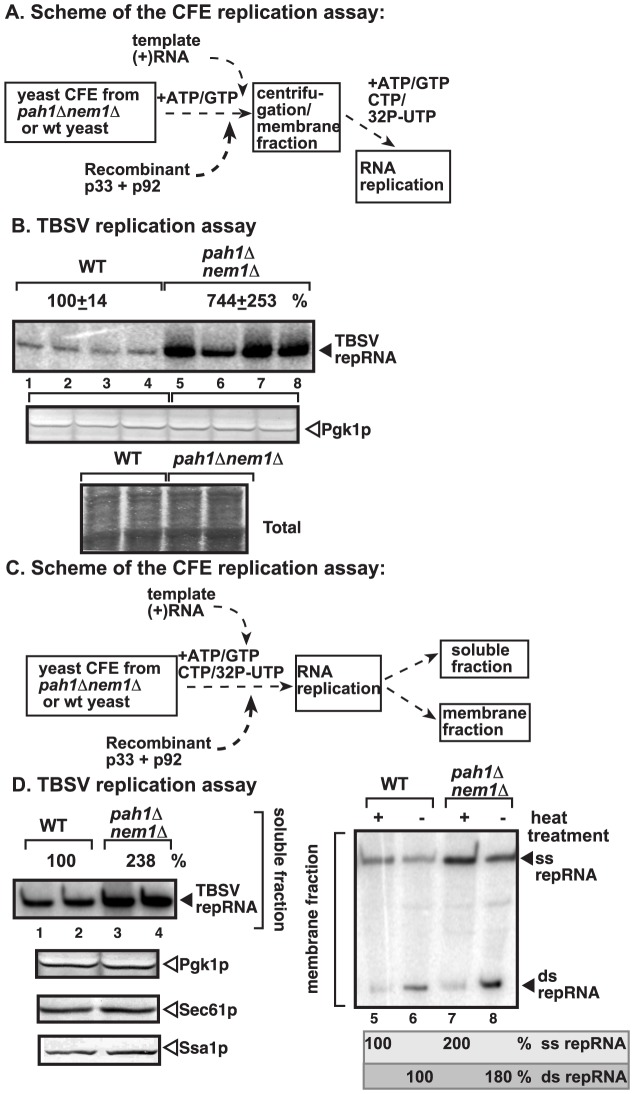
Enhanced TBSV repRNA replication in CFE prepared from *pah1Δ nem1Δ* yeast. (A) The scheme of the CFE-based TBSV replication assay. Purified recombinant TBSV p33 (7 pmol) and p92^pol^ (4 pmol) replication proteins in combination with DI-72 (+)repRNA (0.5 µg) were added to the CFEs. After the VRC assembly in the presence of rATP and rGTP, the membrane fraction of the CFE was collected by centrifugation and the replication assay was performed in the presence of the shown ribonucleotides. (B) TBSV replication assay based on CFEs prepared from wt (lanes 1–4) or *pah1Δ nem1Δ* yeasts (lanes 5–8). Denaturing PAGE analysis of the ^32^P-labeled repRNA products obtained is shown. The full-length single-stranded repRNA is pointed at by an arrow. Note that, prior to the TBSV replication assay, the CFEs were adjusted to contain comparable amounts of the cellular Pgk1p, a cytosolic protein marker. Bottom image shows coomassie-stainde SDS PAGE of the total proteins in the CFEs. (C) The scheme of the modified CFE-based TBSV replication assay to show full *in vitro* replication. Note that the membrane fraction of the CFEs contain the viral VRCs and the bound TBSV RNAs, while the soluble fraction contains the released (+)RNA after replication. (D) Left panel: The denaturing PAGE analysis of the ^32^P-labeled repRNA products in the soluble fraction of the CFEs prepared from wt (lanes 1–2) or *pah1Δ nem1Δ* yeasts (lanes 3–4) is shown. Right panel: Non-denaturing PAGE analysis of the ^32^P-labeled repRNA products in the membrane fraction of the CFEs prepared from wt (lanes 5–6) or *pah1Δ nem1Δ* yeasts (lanes 7–8) is shown. The full-length single-stranded repRNA and the double-stranded repRNA are pointed at by an arrow. The even numbered lanes represent replicase products, which were not heat-treated (thus both ssRNA and dsRNA products are present), while the odd numbered lanes show the heat-treated replicase products (ssRNA is present). The amount of ssRNA and the ratio of ssRNA/dsRNA in the samples are shown. Note that, in the nondenatured samples, the dsRNA product represents the annealed (−)RNA and the (+)RNA, while the ssRNA products represents the newly made (+)RNA products. Bottom panels show Western blot analysis of the CFEs for Pgk1p, Sec61p and Ssa1p cellular proteins. Note that, prior to the assay, the CFEs were adjusted to contain comparable amounts of the cellular Pgk1p.

Based on the above data, it is possible that TBSV VRCs could assemble more efficiently in *pah1Δnem1Δ* yeast due to the presence of extended ER membranes and abundant amounts of phospholipids. To test this possibility, we utilized an *in vitro* tombusvirus VRC assembly assay based on purified recombinant replication proteins and cell-free extracts (CFE) obtained from *pah1Δnem1Δ* or wt yeast (Fig. 3A). In this assay, the tombusvirus (+)repRNA performs one cycle of asymmetrical replication supported by the tombusvirus VRCs assembled *in vitro* [37], [59]. Since we use recombinant viral proteins and repRNA in this assay, we can make sure that only the CFEs, involving the cellular membranes and possibly host factors, are different. The yeast CFEs were adjusted to contain comparable amounts of Pgk1p (a cytosolic protein) (Fig. 3B) and total proteins.

Interestingly, the *in vitro* RNA replication supported by CFE was ∼7-fold higher when assembled in CFE obtained from *pah1Δnem1Δ* yeast than from wt yeast (Fig. 3B, lanes 5–8 versus 1–4). These data strongly suggest that the tombusvirus replicase assembly in the CFE derived from *pah1Δnem1Δ* is more efficient than in the CFE from wt yeast.

To test if TBSV replication indeed includes a full cycle in the CFE from *pah1Δnem1Δ* yeast, we analyzed the (−) and (+)-strand RNAs in the CFEs (Fig. 3C). The membrane-fraction of the CFE at the end of the replication assay contains both single-stranded (ss)RNA [representing the newly made (+)-stranded progeny RNA] and dsRNA [representing the annealed (−) and (+)RNAs]. In addition, the soluble fraction contains the newly released (+)RNAs from the membrane-bound VRCs. We found that the amounts of both ssRNA and dsRNA were ∼2-fold higher in the membrane fraction and the ssRNA was ∼2-fold higher in the soluble fraction of the CFE prepared from *pah1Δnem1Δ* yeast than from the wt yeast (Fig. 3D). These results suggest that the CFE obtained from *pah1Δnem1Δ* yeast performs all the replication steps more efficiently than the CFE prepared from wt yeast cells.

To test if the membrane-fraction of the CFE from *pah1Δnem1Δ* yeast is important for the enhanced TBSV RNA replication, we separated the soluble and membrane fractions of the CFEs prepared from *pah1Δnem1Δ* and wt yeasts and then used various combinations of these fractions for *in vitro* TBSV replication (Fig. S4A). Interestingly, the CFE containing the mixture of the membrane fraction from *pah1Δnem1Δ* yeast and the soluble fraction from the wt yeast supported almost as efficient *in vitro* TBSV replication as the CFE consisting of both fractions from *pah1Δnem1Δ* yeast (Fig. S4B, compare lanes 3–4 and 7–8 with 1–2). Altogether, it seems that the membrane fraction when derived from *pah1Δnem1Δ* yeast was able to support ∼5-fold higher TBSV replication than the membrane fraction from wt yeast in the CFE-based replication assay. This is not surprising since *PAH1* deletion is expected to dramatically change the ER membranes that could be utilized by TBSV for assembly of the VRCs.

### Tombusvirus replicase utilizes the ER membrane derived from yeast lacking *PAH1* more efficiently than from wt yeast

**Figure 4:**
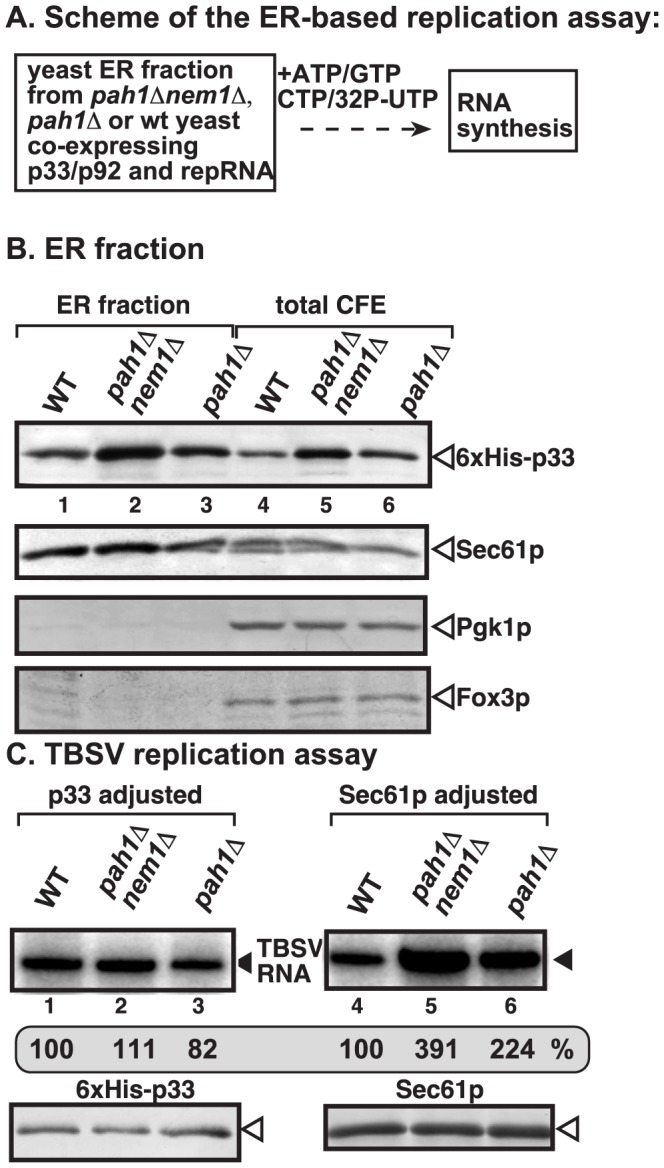
Enhanced TBSV repRNA replication in isolated ER preparations from *pah1Δ* and *pah1Δ nem1Δ* yeasts. (A) The scheme of the CFE-based TBSV replication assay is shown. The isolated ER preparations contained the assembled VRCs (including CNV p33 and p92 replication proteins). The *in vitro* replication assay was performed in the presence of the shown ribonucleotides. (B) Western blot analysis of the isolated ER preparations (lanes 1–3) and the total CFEs (lanes 4–6). The levels of 6×His-p33 (top panel); Sec61p ER marker (second panel); Pgk1p cytosolic protein (third panel), and Fox3p peroxisomal marker (bottom panel) are shown. (C) TBSV replication assay based on isolated ER preparations from wt (lanes 1 and 4), *pah1Δ nem1Δ* (lanes 2 and 5) or *pah1Δ* yeasts (lanes 3 and 6). The ER preparations were normalized based on p33 (lanes 1–3) or the cellular Sec61p (lanes 4–6) prior to the replication assay. The denaturing PAGE analysis of the ^32^P-labeled repRNA products obtained is shown.

To obtain direct evidence that the expanded ER structures and membranes in *pah1Δnem1Δ* yeast are utilized efficiently for TBSV replication, we took advantage of an isolated ER-based tombusvirus replication assay [42]. In this assay, the tombusvirus (+)repRNA can also perform full replication supported by the tombusvirus VRCs assembled in the ER membrane (Fig. 4A) [42]. As expected, the isolated ER preparations contained the Sec61p ER-resident protein, while lacked the cytosolic Pgk1p and the peroxisomal (Fox3p) proteins (Fig. 4B, lanes 1–3 versus 4–6, representing the total CFE). Larger amounts of the p33 replication protein were associated with the ER fraction obtained from *pah1Δnem1Δ* or *pah1Δ* yeasts than from the wt yeast (Fig. 4B, lanes 2 and 3 versus 1), suggesting that p33 utilized the ER membranes more efficiently in yeast lacking the *PAH1* gene.

When we used similar amounts of isolated ER membranes (based on adjusted cellular Sec61p level) for TBSV replication, we observed that the replication of TBSV RNA was 2-to-4-fold higher in ER preparations obtained from *pah1Δnem1Δ* or *pah1Δ* yeasts than from wt yeast (compare lanes 5–6 with 4, Fig. 4C). These data suggest that the ER membranes derived from yeast lacking *PAH1* are more efficiently utilized by the tombusvirus replicase than the ER membrane from wt yeast. On the contrary, when we adjusted the p33 levels in the isolated ER preparations, then we observed comparable levels of *in vitro* tombusvirus replication in ER membranes from all three yeast strains (Fig. 4C, lanes 1–3). This indicates that the relative activity of the tombusvirus replication protein expressed in these yeast strains is similar. Therefore, the higher activity of tombusvirus replicase in *pah1Δnem1Δ* or *pah1Δ* yeasts are likely due to the more efficient assembly of the tombusvirus VRCs, resulting in larger number of replicationally active VRCs than in wt yeast.

**Figure 5:**
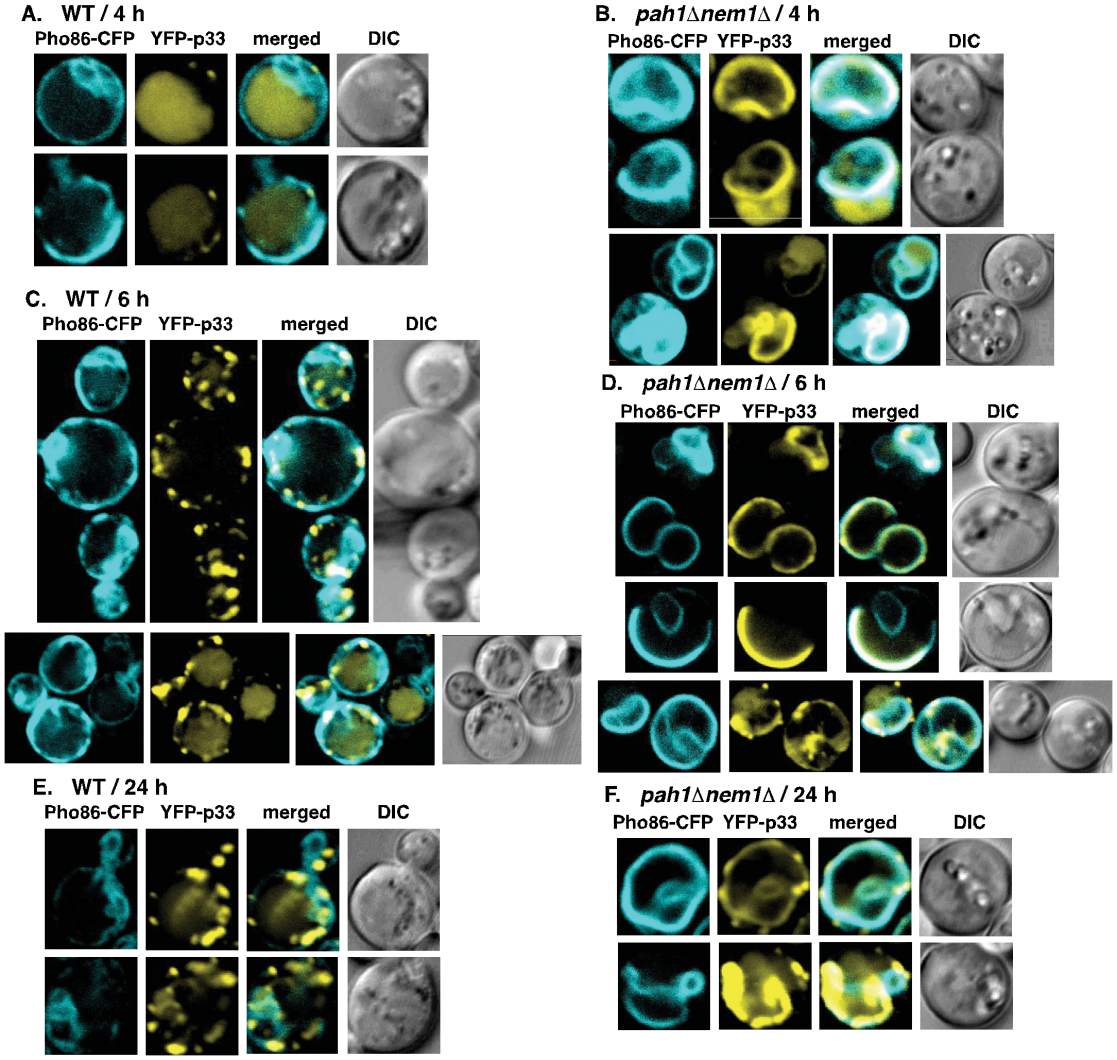
Rapid exploitation of expanded ER membranes by the tombusvirus p33 replication protein in *pah1Δ nem1Δ* yeast. (A) YFP-tagged p33 and CFP-Pho86 (an ER marker) were co-expressed in WT (RS453) or (B) *pah1Δ nem1Δ* yeasts. YFP-p33 was expressed for 1 hour only, followed by additional 3 hours of incubation. The confocal images were taken 4 hours after the induction of YFP-p33 expression at 23°C. (C) YFP-p33 was expressed in RS453 or (D) *pah1Δ nem1Δ* yeasts for 1 hour only, followed by additional 5 hours of incubation prior to imaging. The confocal images were taken 6-hour time point. (E) YFP-p33 was expressed in RS453 or (F) *pah1Δ nem1Δ* yeasts for 24 hours prior to imaging.

To test how efficiently the p33 and p92 replication proteins can utilize the expanded ER membranes in *pah1Δnem1Δ* yeast versus the wt yeast, we performed confocal laser microscopy with fluorescently tagged tombusvirus p33 replication protein. When we looked at the localization of YFP-p33 at an early time point (4 hours), we observed that a large portion of YFP-p33 was still cytosolic and a small number of punctate structures were forming in wt yeast (Fig. 5A). At the same time point, the YFP-p33 localized efficiently in the ER membranes in *pah1Δnem1Δ* yeast and only a smaller fraction of YFP-p33 showed cytosolic localization pattern (Fig. 5B). At the 6 hr time point, the YFP-p33 also showed mostly punctate pattern in wt yeast (Fig. 5C). A fraction of YFP-p33 was present in the ER membrane or was diffused in the cytosol, while other YFP-p33 molecules were likely localized in the peroxisomal membranes in wt yeast. In contrast, most YFP-p33 localized in the ER membranes in *pah1Δnem1Δ* yeast at the 6 h time point (Fig. 5D). At the late 24 h time point, as expected, most of the p33 was localized in punctate structures separate from the ER membranes [likely representing the peroxisomal membranes as shown previously [39]–[41]], although a small fraction of p33 did co-localize with the ER in wt yeast cells (Fig. 5E). In contrast, most p33 is localized in the ER membranes, forming large elongated structures in *pah1Δnem1Δ* yeast (Fig. 5F). Altogether, these data showed that p33 is rapidly localized to the expanded ER membranes in *pah1Δnem1Δ* yeast, while the localization of p33 from the cytosol to membranes is slower in wt yeast, and likely involves the peroxisomal membranes as shown before. It seems that a small fraction of p33 does target the ER membrane even in the wt yeast cells. Therefore, we conclude that the tombusvirus replication proteins efficiently exploit the expanded ER membranes in *pah1Δnem1Δ* yeast cells.

**Figure 6:**
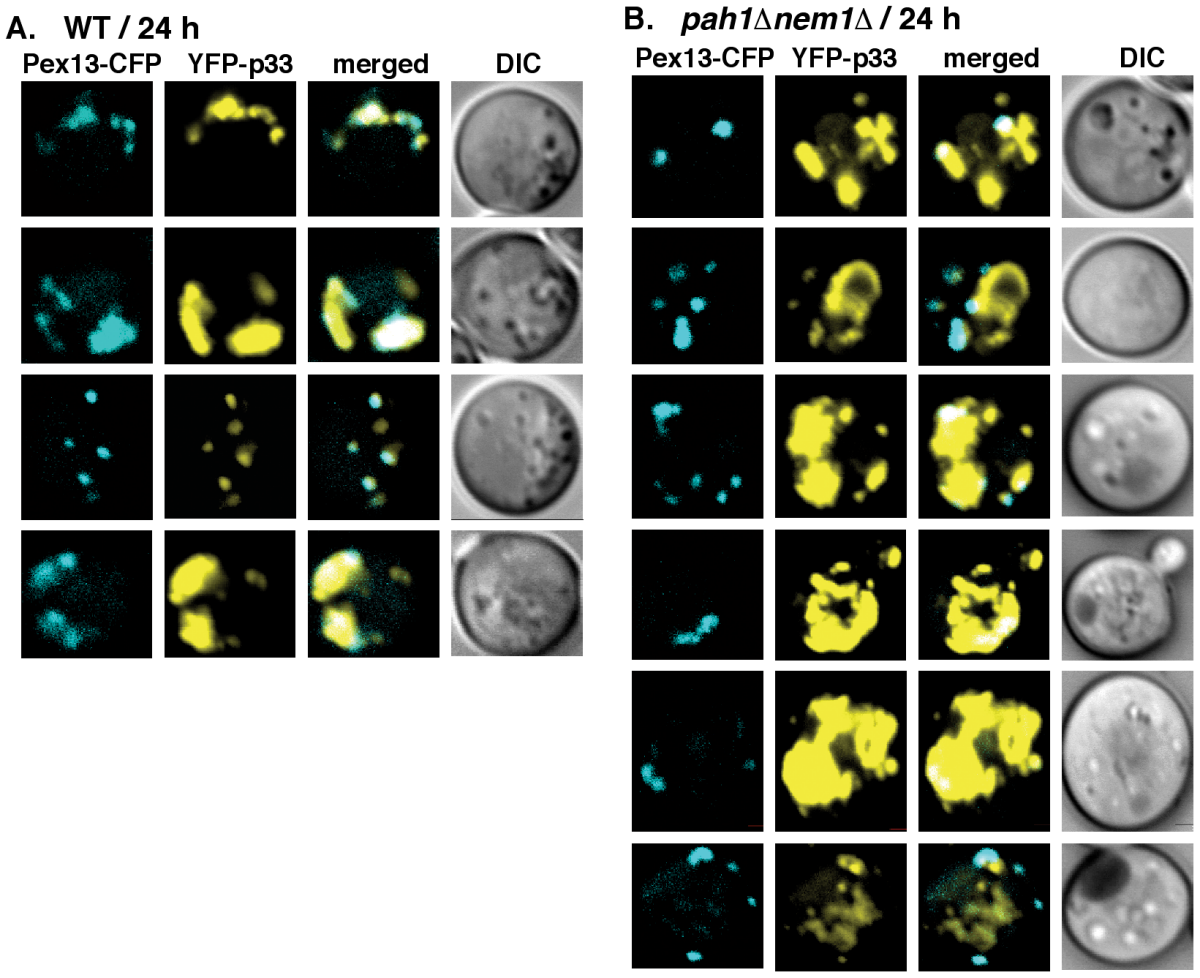
Only a small portion of the tombusvirus p33 replication protein co-localize with peroxisomes in *pah1Δ nem1Δ* yeast. (A) YFP-tagged p33 and CFP-Pex13 (a peroxisomal membrane marker) were co-expressed in WT (RS453) or (B) *pah1Δ nem1Δ* yeasts. The confocal images were taken 24 hours after the induction of YFP-p33 expression at 23°C. See further details in Fig. 5.

To test if peroxisomes are available for TBSV replication in *pah1Δnem1Δ* yeast cells, we used CFP-tagged Pex13p peroxisome membrane marker protein [41]. The distribution of Pex13p showed the characteristic punctate structures in *pah1Δnem1Δ* yeast cells (Fig. 6). However, the co-localization of Pex13p and the tombusvirus p33 differed in wt and in *pah1Δnem1Δ* yeast cells (Fig. 6A–B). While Pex13p showed remarkably good co-localization with the tombusvirus p33 in wt yeast (67% co-localization and 28% partial co-localization of Pex13p and p33 puncta and only 15% of p33 puncta were not co-localized with Pex13p puncta; Fig. 6A), the tombusvirus p33 was largely present in different compartment than Pex13p in *pah1Δnem1Δ* yeast cells (Fig. 6B). However, a small fraction of p33 (less than 10%) was completely or partially co-localized with Pex13p in *pah1Δnem1Δ* yeast cells, suggesting that peroxisome membranes are still utilized by tombusviruses in the mutant yeast. In addition, both Pex13p and the tombusvirus p33 were present in several large foci in wt yeast, indicative of membrane proliferation and peroxisome aggregation induced by the tombusvirus p33 [40], [41], [60]. In contrast, Pex13p was mostly present in several smaller foci in *pah1Δnem1Δ* yeast cells, suggesting lack of membrane proliferation and peroxisome aggregation (Fig. 6B). Altogether, these data is compatible with the model that the expanded ER membranes are more efficiently utilized by the tombusvirus replication protein than the peroxisomal membranes in *pah1Δnem1Δ* yeast cells, although peroxisomes are also available in these cells.

**Figure 7:**
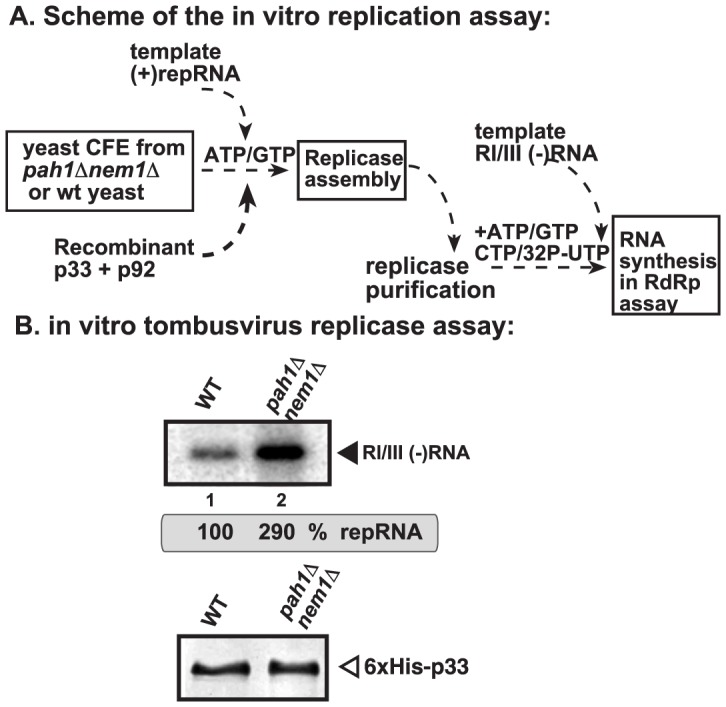
Enhanced activity of the affinity-purified tombusvirus replicase assembled in CFE from *pah1Δ nem1Δ* yeast. (A) Scheme of the tombusvirus replicase assay. CFEs were prepared from wt and *pah1Δ nem1Δ* yeasts, followed by the addition of purified recombinant tombusvirus p33 and p92^pol^ replication proteins and DI-72 (+)repRNA in the presence of rATP and rGTP. The *in vitro* assays were programmed with RI/III (−)RNA in the presence of rATP/rCTP/rGTP and ^32^P-rUTP. (B) Top panel: Representative denaturing gel of ^32^P-labeled RNA products synthesized by the purified tombusvirus replicase *in vitro*. Each experiment was repeated. Bottom panel: Western blot analysis of p33 in the shown replicase samples.

To test if the VRC assembly could indeed be more efficient in the *pah1Δnem1Δ* yeast cells, we used the CFE-based assay to assemble the membrane-bound VRCs [37]. After the assembly and activation of the VRCs in the CFEs, we solubilized and affinity-purified the tombusvirus replicase and tested the replicase activity on added RNA template (Fig. 7A). This assay depends on the efficiency of VRC assembly and the activation of the tombusvirus p92^pol^ replication protein, which is originally inactive when expressed in *E. coli*, yeast or plants [31]. The activation of p92^pol^ replication protein occurs in the membrane-bound VRCs and depends on many factors, including p33, *cis*-acting elements in the viral (+)RNA, host factors and cellular membranes [30], [32], [37], [61], [62]. We found that the CFE prepared from *pah1Δnem1Δ* yeast cells resulted in ∼3-fold higher replicase activity *in vitro* (Fig. 7B), suggesting that the VRC assembly and activation of p92^pol^ replication protein is more efficient than that in the similar CFE from wt yeast.

### CIRV and NoV replicating in the mitochondrial membranes are not benefitted from the expanded ER membranes in yeast lacking *PAH1*

**Figure 8:**
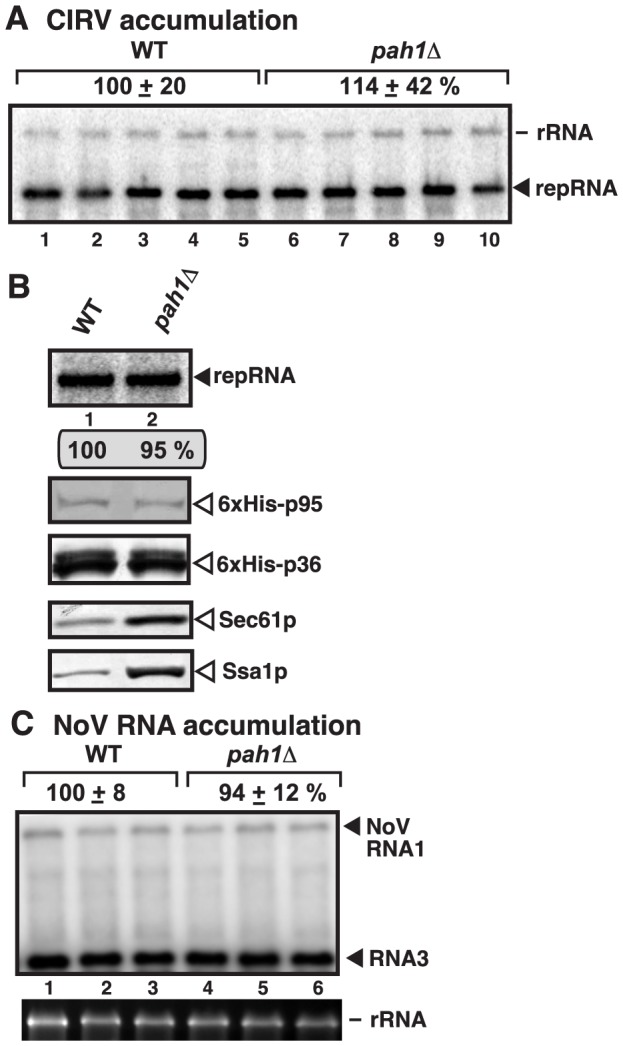
Deletion of *PAH1* does not affect the accumulation of CIRV and NoV RNAs in yeast. (A) Top panel: Replication of the repRNA supported by the CIRV p36 and p95 replication proteins in wt and *pah1Δ* yeast was measured by Northern blotting 48 h after initiation of replication. The accumulation level of repRNA was normalized based on the rRNA. Each sample is obtained from different yeast colonies. (B) Top panel: Comparable level of *in vitro* replication of repRNA by the CIRV VRCs in the isolated membrane fraction from *pah1Δ* yeast when compared with that from wt yeast. Note that the levels of His_6_-p95 replication protein were normalized as shown in the second panel. The third, fourth and fifth panels show the accumulation levels of His_6_-p36, and the cellular Sec61p (an ER marker) and Ssa1p Hsp70, based on Western blotting. (C) To launch NoV RNA1 replication, we expressed NoV RNA1 from the copper-inducible *CUP1* promoter in the parental (BY4741) and in *pah1Δ* yeast strains. Northern blot analysis was used to detect NoV RNA1 and the subgenomic RNA3 accumulation. The accumulation level was normalized based on 18S rRNA. Each experiment was repeated.

To study if other RNA viruses that replicate in the mitochondrial membranes could take advantage of the expanded ER membranes or increased phospholipid synthesis in *pah1Δ* yeast, first we used *Carnation Italian ringspot virus* (CIRV) (Table S1), a tombusvirus closely related to TBSV [29]. CIRV replicates on the outside surface of the mitochondrial membranes *in vivo* and *in vitro* [42], [63], [64]. Interestingly, the level of CIRV replication was not changed in *pah1Δ* yeast (Fig. 8A, lanes 6–10). Also, the membrane-fraction obtained from *pah1Δ* yeast resulted in similar level of RNA replication supported by the CIRV p36 and p95^pol^ replication proteins than the membrane-fraction from wt yeast (Fig. 8B, lane 2 versus 1). The accumulation levels of p36 and p95^pol^ were comparable in *pah1Δ* yeast, while the accumulation of cellular Sec61p and Ssa1p was increased as expected (Fig. 8B). Based on these data, we suggest that CIRV replication is not affected in yeast lacking the *PAH1* gene and the expanded ER membranes do not seem to be utilized by CIRV in yeast.

The second RNA virus tested was Nodamura virus (NoV) (Table S1), an insect RNA virus not related to tombusviruses. NoV RNA replicates on the outer mitochondrial membranes in yeast cells by expressing a single replication protein termed protein A [65]–[67]. We found similar level of NoV RNA accumulation in *pah1Δ* and wt yeasts (Fig. 8C), suggesting that NoV does not take advantage of the cellular changes caused by the deletion of *PAH1*.

### Over-expression of *Arabidopsis* Pah2p interferes with tombusvirus replication in *Nicotiana bethamiana*

To obtain evidence if the conserved lipin-like PAP phosphatidate phosphatase also plays a role in tombusvirus replication in plants, we over-expressed the *Arabidopsis* Pah2p protein in *N. benthamiana* leaves using an *Agrobacterium*-based expression system. Plants have two phosphatidate phosphatase-coding genes, *PAH1* and *PAH2*, which are highly homologous with the yeast *PAH1* gene and they can complement the phenotypes in *pah1Δ* yeast [68]–[70]. Also, deletion of *PAH1* and *PAH2* in *Arabidopsis thaliana* causes ER expansion and increased phospholipid synthesis, similar to the phenotypes observed in *pah1Δ* yeast [69].

**Figure 9:**
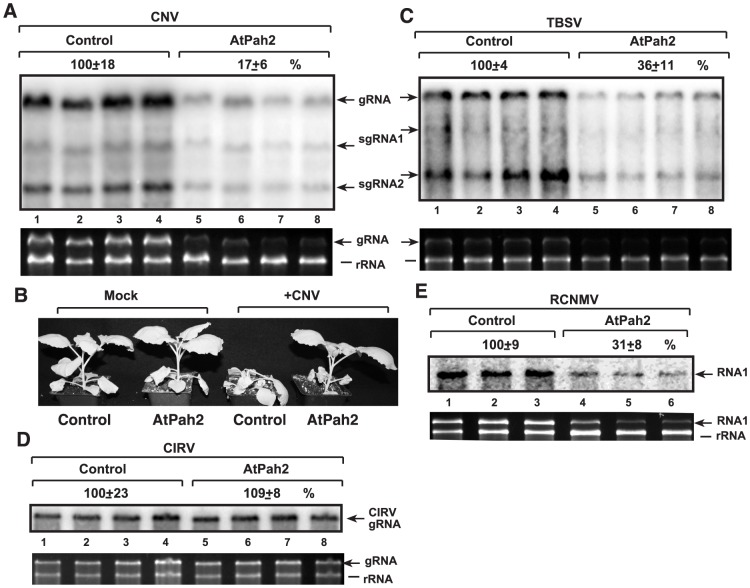
Inhibition of tombusvirus and RCNMV RNA accumulation in plants by over-expression of AtPah2p in *N. benthamiana*. (A) Expression of the yeast *PAH1* homolog AtPah2p (lanes 5–8) was done in *N. benthamiana* leaves by agroinfiltration. Two days later, the same leaves were infiltrated with *Agrobacterium* carrying a plasmid to launch CNV replication from the 35S promoter. The control samples were obtained from leaves expressing no proteins (lanes 1–4). Total RNA was extracted from leaves 5 days after agroinfiltration that launched CNV replication. The accumulation of CNV gRNA and subgenomic (sg)RNAs in *N. benthamiana* leaves was measured by Northern blotting (Top panel). The ribosomal RNA (rRNA) was used as a loading control and shown in agarose gel stained with ethidium-bromide (Second panel). (B) Over-expression of AtPah2p in *N. benthamiana* protects the plant from rapid necrosis caused by systemic CNV infection. The pictures were taken 12 days after agroinfiltration. (C) Inhibition of the peroxisomal TBSV replication by over-expression of AtPah2p in *N. benthamiana*. The agro-infiltrated leaves were inoculated with TBSV two days later, followed by sampling of the same leaves after 3 day of incubation. The accumulation of TBSV gRNA and subgenomic (sg)RNAs in *N. benthamiana* leaves was measured by Northern blotting. See additional details in panel A. (D) The lack of inhibition of the mitochondrial CIRV tombusvirus by over-expression of AtPah2p in *N. benthamiana*. See additional details in panel A. (E) Inhibition of the distantly related RCNMV (which uses ER membranes for replication) by over-expression of AtPah2p in *N. benthamiana*. The agro-infiltrated leaves were inoculated with RCNMV two days later, followed by sampling of the same leaves after 3 day of incubation. The accumulation of RCNMV RNA1 in *N. benthamiana* leaves was measured by Northern blotting. See additional details in panel A.

We found that the over-expression of AtPah2p caused a 6-fold drop in the genomic RNA accumulation of CNV, a TBSV-like tombusvirus, which replicates in the peroxisomal membrane [39], in *N. benthamiana* leaves (Fig. 9A). The lethal necrotic effect of the CNV tombusvirus was also attenuated in *N. benthamiana* expressing the AtPah2p (Fig. 9B). The accumulation of TBSV RNA also decreased by ∼3-fold in *N. benthamiana* leaves over-expressing AtPah2p (Fig. 9C). On the contrary, the replication of another tombusvirus, CIRV (Table S1), which uses the mitochondrial membrane [63], was not changed in *N. benthamiana* leaves over-expressing AtPah2p (Fig. 9D). Based on these observations, we suggest that the plant phosphatidate phosphatase also plays a role in TBSV and CNV tombusvirus replication that can use peroxisomes and ER membranes, but does not affect the replication of the mitochondrial CIRV in plants.

To test if the inhibitory effect of AtPah2p over-expression is specific to peroxisomal tombusviruses, we studied the accumulation of *red clover necrotic mosaic virus* (RCNMV) (Table S1), which is a distantly related RNA virus replicating in the ER membranes of the host cells [71]. We observed that the accumulation of RCNMV RNA decreased by ∼3-fold in *N. benthamiana* leaves over-expressing AtPah2p (Fig. 9E). Thus, another plant RNA virus, RCNMV, is also affected by the plant lipin-like gene, suggesting that the effect of this host gene on ER membranes is critical for replication of RNA viruses taking advantage of the ER membrane to build replication complexes.

## Discussion

Tombusviruses, like many (+)RNA viruses, depend on host membrane biogenesis during replication. Thus, conditions that induce membrane biogenesis in cells might affect replication of some RNA viruses. Accordingly, the key finding in this paper is that tombusviruses (TBSV and CNV) can take advantage of expanded ER membrane surface, which is due to deletion of the cellular Pah1p PAP enzyme leading to massive enlargement of ER membranes and increased phospholipid biosynthesis in yeast [43], [49], [55], [72], to build VRCs efficiently. The evidence supporting this model is extensive and includes: (i) increased TBSV repRNA accumulation in *pah1Δ* (or in the functionally similar *pah1Δnem1Δ* lipin deficient) yeasts or Dgk1p diacylglycerol kinase over-producing yeast; (ii) increased accumulation and stability of tombusvirus p33 replication protein in *pah1Δ* yeast; (iii) the enhanced *in vitro* assembly of the tombusvirus VRCs in a CFE-based assay derived from yeast lacking Pah1p; (iv) detection of highly abundant p33 replication proteins in isolated ER fraction or using confocal microscopy in yeast lacking Pah1p; (v) the higher *in vitro* activity of the tombusvirus replicase purified from CFE obtained from yeast lacking Pah1p than from wt yeast; and (vi) the stimulatory role of the membrane fraction of *pah1Δ* yeast in the CFE-based TBSV replication assay. The efficient utilization of the expanded ER membrane is reflected by the rapid localization of the p33 replication protein to the ER membrane at an early time point in yeast lacking Pah1p. In addition, it appears that the tombusvirus replicase assembles faster in yeast lacking Pah1p than in wt yeast. Thus, our model proposes that TBSV and CNV efficiently subvert the expanded ER membranes and utilizes the abundant phospholipids generated in *pah1Δ* or *pah1Δnem1Δ* lipin-deficient yeast, leading to robust viral replication. These tombusviruses replicate in the expanded ER membranes in lipin deficient yeast although the peroxisomal membranes are also present (and some of these membranes are still utilized by TBSV and CNV), suggesting that the expanded ER likely contains favorable microenvironment (membrane microdomains) for these tombusviruses. Therefore, it seems that TBSV and CNV are flexible in utilizing peroxisomal and ER membranes depending on cellular conditions. Accordingly, TBSV is capable to assemble the VRCs on ER and mitochondrial preparations *in vitro* [42], indicating that TBSV could exploit a range of subcellular membranes in cells. Altogether, we document that deletion of the lipin gene results in strong stimulatory effect on TBSV replication. We also show that TBSV could readily switch to the vastly expanded ER membranes in lipin-deficient cells to build VRCs and support robust viral replication instead of utilizing the peroxisomal membranes as observed in wt yeast and plants [5], [39]. Thus, the increased phospholipid synthesis and the expanded ER membranes in lipin-deficient cells provide highly suitable environment for TBSV and CNV for efficient viral replication.

Similar to tombusviruses, other (+)RNA viruses subvert various intracellular membranes for construction of VRCs, and the ER membrane is often critical for these processes [4], [73]–[75]. Indeed, we find that the ER-based RCNMV replication in plant host cells is also affected by the lipin gene. Therefore, our findings with tombusviruses presented in this paper could be relevant for other RNA viruses of plants and animals. However, not all viruses could benefit from lipin mutations, since we find that CIRV tombusvirus and NoV insect RNA virus, both of which replicate on the mitochondrial membrane surfaces, could not take advantage of the expanded ER membranes generated in *pah1Δ* yeast. CIRV was also more restrictive in the CFE-based replication assay, utilizing the mitochondrial membranes more efficiently than the ER membranes [42].

The role of *PAH1* in tombusvirus replication based on yeast model host was further supported by data obtained in natural plant host by over-expression of AtPah2p. The over-expression of the yeast Pah1p (especially the constitutively active Pah1-7A mutant) in yeast and the AtPah2p in *N. benthamiana* strongly inhibited tombusvirus replication. This inhibition is likely due to strong competition between cellular phospholipid pathways driven by the over-expressed PAP enzyme and the need for phospholipid-containing cellular membranes in tombusvirus replication [76]. We suggest that tombusviruses could find and/or induce phospholipid-rich microdomains in peroxisomal or ER membranes less efficiently when the over-expressed cellular PAP enzyme decreases the phosholipids level (by producing DAG) in cells.

Our findings might also influence the current view on genetic mutations and diseases versus pathogen infections. Mutations in cellular genes frequently alter cellular pathways and they can cause genetic diseases. The same gene mutations/changes might also influence pathogen-host interactions as well. For example, the cellular lipin, which is involved in a key cellular decision on using lipids for membrane biogenesis or for storage [43]–[47], could be one of those factors. We suggest that mutations in lipin genes, which are known to induce many genetic diseases in humans and animals [44], [46], [47], [77]–[79], not only change the physiology of the given organism, but they might affect pathogens and their interactions with the altered host. Although we used yeast as a model host, since it only has a single lipin gene (*PAH1*), the role of the PAP enzyme in phospholipid pathways are conserved in yeast, plants and animals. Deletion/mutations of the lipin gene is known to facilitate membrane biogenesis in all these organisms. Therefore, the effects seen with tombusviruses and RCNMV might also manifest with animal viruses possibly leading to exploitation of the expanded ER membranes during infections. Thus, it is possible that one disease state might facilitate the development of robust viral infection at the cellular level.

## Materials and Methods

### Yeast strains and expression plasmids

*Saccharomyces cerevisiae* strain RS453 (*MATa ade2-1 his3, 15 leu2-3, 112 trp1-1 ura3-52*), pah1Δ (pah1Δ::TRP1 derivative of RS453) and pah1Δnem1Δ (pah1Δ::TRP1 nem1Δ::HIS3 derivative of RS453) were published previously [53].

We found that the pah1Δnem1Δ yeast was more consistent than pah1Δ yeast in supporting high level of CNV and TBSV repRNA replication for yet unknown reasons. Therefore, most of the experiments were performed with both strains or with only pah1Δnem1Δ yeast strain.

The yeast expression plasmids, pYEplac181, pYEplac181-wt-Pah1 and pYEplac181-Pah1-7A have been obtained from Dr. George M. Carman [53]. The following yeast expression plasmids have been prepared before: LpGAD-CUP1-HisFlag-p92 (*LEU2* selection), UpGBK-ADH-His-p33/GAL1-DI-72 (*URA3* selection), HpESC-GAL1-His-p33/GAL10-DI-72 (*HIS3* selection) [80]; HpGBK-CUP1-HisFlag-p33/GAL1-DI-72 (*HIS3* selection) [81]; UpESC-CUP1-His-p92 (*URA3* selection) [82]; UpYES-GAL-Hisp33 (URA3 selection) [76], UpESC-YFP-p33 (*URA3* selection), LpGAD-Pho86-CFP (*LEU2* selection) [39]; UpESC-GAL1-C36/GAL10-DI-72 (*URA3* selection), HpYES-GAL1-C95 (*HIS3* selection) [42]. The plasmids pMAL-33, pMAL92 and pET-His-MBP-p33 expressing CNV viral proteins in *E. coli* were described earlier [61], [83]. LpGAD-ADH::Pex13-CFP (*LEU2* selection) [39]; UpESC-GAL1::C36/GAL10::DI-72 (*URA3* selection), HpYES-GAL1::C95 (*HIS3* selection), UpYES-GAL1::T92 (*URA3* selection), HpESC-GAL1::T33/ GAL10::DI-72 (*HIS3* selection) [42].

### Pah1p complementation and over-expression assays in yeast

Yeast wt RS453 and *pah1Δ* strain was transformed with pESC-CUP1-His-p92 (URA3 selection), pESC-GAL1-His-p33/GAL10-DI-72 (*HIS3* selection) and *LEU2* based plasmids: pYEplac181 (vector control), pYEplac181-wt-Pah1p (for expression of wt Pah1p) or pYEplac181-Pah1p-7A (for expression of constitutively active phosphorylation-deficient Pah1p), respectively [53]. Yeast was pre-grown at 23°C overnight in 2 ml SC-ULH^−^ medium containing 2% galactose and then the cultures were harvested after 24 h 50 µM CuSO_4_ induction.

### Replication protein stability assay

To study the stability of p33 replication protein in yeast, RS453 and *pah1Δnem1Δ* strains were transformed with plasmid UpYES-GAL-Hisp33 expressing His_6_-tagged CNV p33 from the galactose-inducible *GAL1* promoter. Yeast transformants were cultured overnight in SC-U^−^ medium containing 2% glucose at 23°C. Yeast cultures were transferred to SC-U^−^ medium supplemented with 2% galactose for 3 h at 23°C. Then, the cultures were shifted back to the SC-U^−^ medium supplemented with 2% glucose and cycloheximide (at a final concentration of 100 µg/ml). The amount of p33 was detected by Western blotting with anti- His_6_ antibody at given time points after cycloheximide treatment. Each sample loading was adjusted based on total protein levels as determined by SDS-PAGE [39].

### TBSV and CIRV replication assays in yeast

Replication assays were performed by measuring the accumulation of DI-72(+) repRNA relative to the accumulation of the cellular 18S rRNA. For the CNV replication proteins-based replication assay, RS453 (wt), *pah1Δ* or *pah1Δnem1Δ* yeast cells [53] were transformed with plasmid LpGAD-CUP1-HisFlag-p92 and UpGBK-ADH-His-p33/GAL1-DI-72. Then, yeast was pre-grown at 23°C overnight in 2 ml SC-LU^−^ (synthetic complete dropout medium lacking leucine and Uracil) medium containing 2% galactose. Replication of TBSV repRNA was induced by adding 50 µM CuSO_4_ into the medium and, then, the samples were harvested at different time points. The TBSV replication proteins-based assay was similar to that described for CNV above, except yeasts were transformed with plasmids UpYES-GAL1::T92 and HpESC-GAL1:: T33/GAL10::DI-72 were directly grown in 2 ml SC-UH^−^ medium containing 2% galactose for 24 h at 23°C. For CIRV replication assay, yeast strains were transformed with plasmid UpESC-GAL1-C36/GAL10-DI-72 and HpYES-GAL1-C95 and then the yeast was grown at 23°C in 2 ml SC-UH- medium containing 2% galactose for 2 days. Standard RNA extraction and Northern blot analysis was performed as described previously [31], [84].

### In vitro replication assay using yeast membrane fractions

The membrane fractions were prepared as described previously [85]. Briefly, yeasts were transformed and cultured as described in the main text for TBSV or CIRV repRNA replication in yeast. Cultures were collected by centrifugation to obtain membrane fractions containing the *in vivo*-assembled tombusvirus replicase complexes. Each membrane fraction preparation was adjusted by the relative amounts of His_6_-tagged p92 and comparable amounts of replicase from each preparation were used in the subsequent replicase assay. The replicase assay was performed as described [85]. The *in vitro* reaction (50 µl) contained 10 µl of the normalized MEFs preparations, 50 mM Tris–Cl pH 8.0, 10 mM MgCl_2_, 10 mM DTT, 0.1 U RNase inhibitor, 10 mM ATP, 10 mM CTP, 10 mM GTP and 0.1 µl of ^32^P-UTP (3000 Ci/mmol). Reaction mixtures were incubated for 3 h at 25°C, followed by phenol/chloroform extraction and isopropanol/ammonium acetate (10∶1) precipitation. The ^32^P-UTP-labeled RNA products were analyzed in 5% acrylamide/8 M urea gels.

### In vitro replication assay using yeast cell free extract (CFE)

CEFs from RS453 (wt) or *pah1Δnem1Δ* were prepared as described earlier [37] and adjusted to contain comparable amounts of cellular Pgk1p, a cytosolic protein marker. The *in vitro* reaction was performed in 20 µl total volume containing 2 µl of adjusted CFE, 0.5 µg DI-72 (+)repRNA transcript, 0.5 µg purified MBP-p33, 0.5 µg purified MBP-p92^pol^ (both recombinant proteins were purified from *E. coli*), 30 mM HEPES-KOH, pH 7.4, 150 mM potassium acetate, 5 mM magnesium acetate, 0.13 M sorbitol, 0.4 µl actinomycin D (5 mg/ml), 2 µl of 150 mM creatine phosphate, 0.2 µl of 10 mg/ml creatine kinase, 0.2 µl of RNase inhibitor, 0.2 µl of 1 M dithiothreitol (DTT), 2 µl of 10 mM ATP, CTP, and GTP and 0.25 mM UTP and 0.1 µl of ^32^P-UTP. Reaction mixtures were incubated 3 h at 25°C, followed by phenol/chloroform extraction and isopropanol/ammonium acetate (10∶1) precipitation. ^32^P-UTP-labeled RNA products were analyzed in 5% acrylamide/8 M urea gels [37]. To detect the double-stranded RNA (dsRNA) in the cell-free replication assay, the ^32^P-labeled RNA samples were directly loaded onto the gel without heat treatment. Membrane and soluble fractions of these CFEs or in vitro reaction were separated by centrifugation at 35,000 g for 30 min and then mixed them in various combinations (described in the figure legends).

### *In vitro* TBSV replication assay using isolated yeast ER fractions

Yeasts were transformed and cultured as described in the main text for TBSV or CIRV repRNA replication in yeast. The ER fractions were prepared as described earlier [42], [86]. Briefly, yeast cells were made into spheroplasts by incubating with 5 mg/g (wet weight) Zymolyase 20T (Seikagaku), and then the spheroplasts were homogenized and lysed with a glass Dounce homogenizer in ice-cold HEPES lysis buffer (20 mM HEPES/KOH [pH 6.8], 50 mM potassium acetate, 100 mM sorbitol, 2 mM EDTA, 1 mM DTT and 1% [V/V] yeast protease inhibitor cocktail [Ypic]). The homogenized spheroplasts were then centrifuged at 1,000 g for 10 min at 4°C, and the supernatant was subjected to additional centrifugation at 27,000 g for 10 min at 4°C to obtain the membrane preparation. To further purify the ER fraction, the membrane preparation was subjected to centrifugation at 100,000 g on a sucrose step gradients (1.5 M and 1.2 M sucrose/HEPES). The purified ER fraction was recovered between the sucrose gradient interfaces and each fraction was adjusted by the amounts of the protein mentioned in figure legend. The *in vitro* TBSV replication assay was performed as described for in vitro replication assay with MEF except that using the purified ER fraction.

### Affinity-purification of the *in vitro* assembled TBSV replicase

200 µl of the CFE-based replication assay was performed as described above, except that only rATP and rGTP were used. Also, the CFE assay contained the MBP and His_6_-tagged recombinant p33. At the end of the *in vitro* replicase assembly assay, the reaction mixture was diluted with 800 µl solubilization buffer, and the replicase complex was purified followed the procedure described previously [61]. In vitro RdRp activity assay was performed using DI-72 region I/III (−)RNA or region IV (+)RNA as template transcribed in vitro by T7 transcription.

### Imaging yeast cells with confocal laser microscopy

To visualize the ER, Pho86-CFP was used as a marker, while peroxisomes were monitored with the help of Pex13-CFP (a peroxisome membrane marker) [39]. The yeast cells were transformed with UpESC-YFP-p33 and LpGAD-Pho86-CFP or LpGAD-ADH::Pex13-CFP. For the 24 h time point, transformed yeast cells were grown in SC-UL^−^ medium containing 2% galactose at 23°C for 24 h and then sample were collected and analyzed by confocal microscopy as described [41]. For short time points, the transformed yeasts were pre-grown overnight at 23°C in SC-UL^−^ medium containing 2% glucose and then transferred to media containing 2% galactose, and then, samples were collected for microscopy analysis at given time points.

Confocal laser scanning micrographs of yeast cells were acquired on an Olympus FV1000 microscope (Olympus America Inc., Melville, New York) as described [41]. ECFP was excited using 440 nm laser light, attenuated to 4.5% of the maximum laser power, while EYFP was excited using 515 nm laser line (3.5% of the maximum laser power). The images were acquired using sequential line-by-line mode in order to reduce excitation and emission cross-talk. The primary objective used was water-immersion PLAPO60XWLSM (Olympus). Image acquisition was conducted at a resolution of 512×512 pixels and a scan-rate of 10 µs/pixel. Image acquisition and exportation of TIFF files were controlled by using Olympus Fluoview software version 1.5.

### Western blotting

To prepare the total protein sample for Western blotting, we followed a previous protocol [31], [39]. Briefly, 1 ml of yeast culture was harvested by centrifugation. Then, the samples were re-suspended in 200 µl of 0.1M NaOH and incubated at room temperature with shaking for 20 min. The supernatant was removed after a short centrifugation, and the pellet was re-suspended in 50 µl, 1X SDS-polyacrylamide gel electrophoresis (PAGE) buffer containing 5% β-mercaptoethanol and incubated at 85°C for 15 min. The supernatant was used for SDS/PAGE and Western blot analysis as described [87]. To detect the CNV or TBSV viral proteins, anti-His_6_ antibody was used as the primary antibody (Invitrogen) and the secondary antibody was alkaline-phosphatase conjugated anti-mouse IgG (Sigma). For cellular protein markers, the following antibodies were used: anti-3-phosphoglycerate kinase (anti-PGK), and anti-heat shock protein 70 (anti-Hsc70) (purchased from Invitrogen, CA). Sec61p antibody was provided by Tom Rapoport, Harvard Medical School. Fox3p antibody was provided by Daniel J. Klionsky, University of Michigan.

### TBSV and CIRV replication in *Nicotiana benthamiana*

Transient expression of *Arabidopsis thaliana* Pah2p in *N. benthamiana* leaves was performed by agroinfiltration [88]. *A. thaliana PAH2* (At5g42870) was amplified by PCR using primers #4630 (GCCGGATCCATGAATGCCGTCGGTAGGATC) / #4631 (CGGCTCGAGTCACATAAGCGATGGAGGAGGCAG) and genomic DNA as template. The obtained PCR product was digested with *Bam*HI and *Xho*I, purified and ligated into pGD-L [6] previously digested with *Bam*HI and *Sal*I. The resulting plasmid was transformed into *Agrobacterium tumefaciens* C58C1 [6]. *N. benthamiana* plants were infiltrated with *A. tumefaciens* (OD_600_ = 0.8) carrying pGD-L-PAH2 or the control empty plasmid pGD. Two days later, the same leaves were infiltrated with *A. tumefaciens* (OD_600_ = 0.2) carrying pGD-CNV to launch CNV replication, or pGD-CIRV to launch CIRV replication [6]. Leaf samples were collected 5 days later and total RNA was extracted. CNV and CIRV RNA accumulation was analyzed by agarose gel electrophoresis and Northern blotting using ^32^P-labeled probes complementary to the 3′ end of the viral RNAs [6].

For studies with TBSV and RCNMV, *N. benthamiana* plants were infiltrated with *A. tumefaciens* (OD_600_ = 0.8) carrying pGD-L-PAH2 or the control empty plasmid pGD. Two days later, the same leaves were inoculated with infectious saps containing TBSV or RCNMV virions. Leaf samples were collected 3 days later and total RNA was extracted. RNA accumulation was analyzed by agarose gel electrophoresis and Northern blotting using ^32^P-labeled probes complementary to the 3′ end of the TBSV RNA or RCNMV RNA1 [6].

## Supporting Information

Figure S1 Over-expression of Dgk1p enhances TBSV repRNA accumulation in wt yeast as shown by Northern blotting. Note that overproduction of Dgk1p, similar to *PAH1* deletion, leads to ER membrane expansion. Bottom panel: detection of the overproduced Dgk1p in yeast. The His_6_-tagged Dgk1p, His_6_-p33 and His_6_-p92 were detected by Western blotting.
(EPS)

Figure S2 Comparable effects of single *PAH1* and double *PAH1* and *NEM1* deletions on TBSV repRNA accumulation in yeast. Top panel: Replication of the TBSV repRNA in wt, *pah1Δnem1Δ* and *pah1Δ* yeasts was measured by Northern blotting 8 h after initiation of TBSV replication. The accumulation level of repRNA was normalized based on the ribosomal (r)RNA. Each sample is obtained from different yeast colonies. Bottom panel: The accumulation levels of His_6_-p92 and His_6_-p33 were tested by Western blotting. Each experiment was repeated.
(EPS)

Figure S3 Comparable effects of single *PAH1* and double *PAH1* and *NEM1* deletions on *in vitro* TBSV repRNA replication. (A) The scheme of the *in vitro* TBSV replication assay based on the isolated membrane fraction carrying the tombusvirus replicase and the bound RNA template. (B) Top panel: Comparable *in vitro* replication of TBSV repRNA in the isolated membrane fraction from *pah1Δ* yeast when compared with that from *pah1Δ nem1Δ* yeast. Note that the levels of His_6_-p92 replication protein were normalized as shown in the second panel. The third, fourth and fifth panels show the accumulation levels of His_6_-p33, and the cellular Sec61p (an ER marker) and Ssa1p Hsp70. Samples were taken at 24 h time point.
(EPS)

Figure S4 Enhanced TBSV replication depends on the membrane fraction of CFE prepared from *pah1Δnem1Δ* yeast. (A) The scheme of the CFE-based TBSV replication assay. Purified recombinant TBSV p33 (7 pmol) and p92^pol^ (4 pmol) replication proteins in combination with DI-72 (+)repRNA (0.5 µg) were added to the CFEs made by mixing the soluble and membrane fractions as shown. (B) Denaturing PAGE analysis of the ^32^P-labeled repRNA products obtained is shown. The TBSV replication assays were based on the mixed soluble and membrane fractions of CFEs prepared from wt or *pah1Δnem1Δ* yeasts, as shown. The full-length single-stranded repRNA is pointed at by an arrow.
(EPS)

Table S1 Viruses used in this study.
(DOC)

## References

[1] den Boon JA, Diaz A, Ahlquist P (2010) Cytoplasmic viral replication complexes. Cell Host Microbe 8: 77–85. 20638644 10.1016/j.chom.2010.06.010 PMC2921950

[2] Miller S, Krijnse-Locker J (2008) Modification of intracellular membrane structures for virus replication. Nat Rev Microbiol 6: 363–374. 18414501 10.1038/nrmicro1890 PMC7096853

[3] Nagy PD, Pogany J (2012) The dependence of viral RNA replication on co-opted host factors. Nature Reviews Microbiology 10: 137–149. 10.1038/nrmicro2692 PMC7097227 22183253

[4] Belov GA, van Kuppeveld FJ (2012) (+)RNA viruses rewire cellular pathways to build replication organelles. Curr Opin Virol 2: 740–747. 23036609 10.1016/j.coviro.2012.09.006 PMC7102821

[5] McCartney AW, Greenwood JS, Fabian MR, White KA, Mullen RT (2005) Localization of the tomato bushy stunt virus replication protein p33 reveals a peroxisome-to-endoplasmic reticulum sorting pathway. Plant Cell 17: 3513–3531. 16284309 10.1105/tpc.105.036350 PMC1315385

[6] Barajas D, Jiang Y, Nagy PD (2009) A Unique Role for the Host ESCRT Proteins in Replication of Tomato bushy stunt virus. PLoS Pathog 5: e1000705. 20041173 10.1371/journal.ppat.1000705 PMC2791863

[7] Novoa RR, Calderita G, Arranz R, Fontana J, Granzow H, et al (2005) Virus factories: associations of cell organelles for viral replication and morphogenesis. Biol Cell 97: 147–172. 15656780 10.1042/BC20040058 PMC7161905

[8] Salonen A, Ahola T, Kaariainen L (2005) Viral RNA replication in association with cellular membranes. Curr Top Microbiol Immunol 285: 139–173. 15609503 10.1007/3-540-26764-6_5 PMC7120253

[9] Bartenschlager R, Cosset FL, Lohmann V (2010) Hepatitis C virus replication cycle. Journal of Hepatology 53: 583–585. 20579761 10.1016/j.jhep.2010.04.015

[10] Cherry S, Doukas T, Armknecht S, Whelan S, Wang H, et al (2005) Genome-wide RNAi screen reveals a specific sensitivity of IRES-containing RNA viruses to host translation inhibition. Genes Dev 19: 445–452. 15713840 10.1101/gad.1267905 PMC548945

[11] Kushner DB, Lindenbach BD, Grdzelishvili VZ, Noueiry AO, Paul SM, et al (2003) Systematic, genome-wide identification of host genes affecting replication of a positive-strand RNA virus. Proc Natl Acad Sci U S A 100: 15764–15769. 14671320 10.1073/pnas.2536857100 PMC307642

[12] Serviene E, Jiang Y, Cheng CP, Baker J, Nagy PD (2006) Screening of the yeast yTHC collection identifies essential host factors affecting tombusvirus RNA recombination. J Virol 80: 1231–1241. 16415000 10.1128/JVI.80.3.1231-1241.2006 PMC1346934

[13] Panavas T, Serviene E, Brasher J, Nagy PD (2005) Yeast genome-wide screen reveals dissimilar sets of host genes affecting replication of RNA viruses. Proc Natl Acad Sci U S A 102: 7326–7331. 15883361 10.1073/pnas.0502604102 PMC1129141

[14] Krishnan MN, Ng A, Sukumaran B, Gilfoy FD, Uchil PD, et al (2008) RNA interference screen for human genes associated with West Nile virus infection. Nature 455: 242–245. 18690214 10.1038/nature07207 PMC3136529

[15] Castorena KM, Stapleford KA, Miller DJ (2010) Complementary transcriptomic, lipidomic, and targeted functional genetic analyses in cultured Drosophila cells highlight the role of glycerophospholipid metabolism in Flock House virus RNA replication. BMC Genomics 11: 183. 20236518 10.1186/1471-2164-11-183 PMC2847973

[16] Belov GA, Ehrenfeld E (2007) Involvement of cellular membrane traffic proteins in poliovirus replication. Cell Cycle 6: 36–38. 17245115 10.4161/cc.6.1.3683

[17] Hsu NY, Ilnytska O, Belov G, Santiana M, Chen YH, et al (2010) Viral reorganization of the secretory pathway generates distinct organelles for RNA replication. Cell 141: 799–811. 20510927 10.1016/j.cell.2010.03.050 PMC2982146

[18] Sasvari Z, Nagy PD (2010) Making of Viral Replication Organelles by Remodeling Interior Membranes. Viruses-Basel 2: 2436–2442. 10.3390/v2112436 PMC3185585 21994625

[19] Berger KL, Cooper JD, Heaton NS, Yoon R, Oakland TE, et al (2009) Roles for endocytic trafficking and phosphatidylinositol 4-kinase III alpha in hepatitis C virus replication. Proc Natl Acad Sci U S A 106: 7577–7582. 19376974 10.1073/pnas.0902693106 PMC2678598

[20] Heaton NS, Perera R, Berger KL, Khadka S, Lacount DJ, et al (2010) Dengue virus nonstructural protein 3 redistributes fatty acid synthase to sites of viral replication and increases cellular fatty acid synthesis. Proc Natl Acad Sci U S A 107: 17345–17350. 20855599 10.1073/pnas.1010811107 PMC2951450

[21] Nagy PD, Pogany J (2006) Yeast as a model host to dissect functions of viral and host factors in tombusvirus replication. Virology 344: 211–220. 16364751 10.1016/j.virol.2005.09.017

[22] Rajendran KS, Nagy PD (2006) Kinetics and functional studies on interaction between the replicase proteins of Tomato Bushy Stunt Virus: Requirement of p33:p92 interaction for replicase assembly. Virology 345: 270–279. 16242746 10.1016/j.virol.2005.09.038

[23] Serviene E, Shapka N, Cheng CP, Panavas T, Phuangrat B, et al (2005) Genome-wide screen identifies host genes affecting viral RNA recombination. Proc Natl Acad Sci U S A 102: 10545–10550. 16027361 10.1073/pnas.0504844102 PMC1180806

[24] Nagy PD, Barajas D, Pogany J (2012) Host factors with regulatory roles in tombusvirus replication. Curr Opin Virol 2: 685–692. 10.1016/j.coviro.2012.10.004 23122856

[25] Nagy PD, Pogany J (2010) Global genomics and proteomics approaches to identify host factors as targets to induce resistance against Tomato bushy stunt virus. Adv Virus Res 76: 123–177. 20965073 10.1016/S0065-3527(10)76004-8 PMC7173251

[26] Panaviene Z, Baker JM, Nagy PD (2003) The overlapping RNA-binding domains of p33 and p92 replicase proteins are essential for tombusvirus replication. Virology 308: 191–205. 12706102 10.1016/s0042-6822(02)00132-0

[27] Oster SK, Wu B, White KA (1998) Uncoupled expression of p33 and p92 permits amplification of tomato bushy stunt virus RNAs. J Virol 72: 5845–5851. 9621045 10.1128/jvi.72.7.5845-5851.1998 PMC110387

[28] Scholthof KB, Scholthof HB, Jackson AO (1995) The tomato bushy stunt virus replicase proteins are coordinately expressed and membrane associated. Virology 208: 365–369. 11831721 10.1006/viro.1995.1162

[29] White KA, Nagy PD (2004) Advances in the molecular biology of tombusviruses: gene expression, genome replication, and recombination. Prog Nucleic Acid Res Mol Biol 78: 187–226. 15210331 10.1016/S0079-6603(04)78005-8

[30] Panaviene Z, Panavas T, Nagy PD (2005) Role of an internal and two 3′-terminal RNA elements in assembly of tombusvirus replicase. J Virol 79: 10608–10618. 16051853 10.1128/JVI.79.16.10608-10618.2005 PMC1182651

[31] Panaviene Z, Panavas T, Serva S, Nagy PD (2004) Purification of the cucumber necrosis virus replicase from yeast cells: role of coexpressed viral RNA in stimulation of replicase activity. J Virol 78: 8254–8263. 15254197 10.1128/JVI.78.15.8254-8263.2004 PMC446104

[32] Pogany J, Nagy PD (2012) p33-Independent Activation of a Truncated p92 RNA-Dependent RNA Polymerase of Tomato Bushy Stunt Virus in Yeast Cell-Free Extract. J Virol 86: 12025–12038. 22933278 10.1128/JVI.01303-12 PMC3486448

[33] Monkewich S, Lin HX, Fabian MR, Xu W, Na H, et al (2005) The p92 polymerase coding region contains an internal RNA element required at an early step in Tombusvirus genome replication. J Virol 79: 4848–4858. 15795270 10.1128/JVI.79.8.4848-4858.2005 PMC1069561

[34] Panavas T, Hawkins CM, Panaviene Z, Nagy PD (2005) The role of the p33:p33/p92 interaction domain in RNA replication and intracellular localization of p33 and p92 proteins of Cucumber necrosis tombusvirus. Virology 338: 81–95. 15936051 10.1016/j.virol.2005.04.025

[35] Pogany J, White KA, Nagy PD (2005) Specific binding of tombusvirus replication protein p33 to an internal replication element in the viral RNA is essential for replication. J Virol 79: 4859–4869. 15795271 10.1128/JVI.79.8.4859-4869.2005 PMC1069559

[36] Stork J, Kovalev N, Sasvari Z, Nagy PD (2011) RNA chaperone activity of the tombusviral p33 replication protein facilitates initiation of RNA synthesis by the viral RdRp in vitro. Virology 409: 338–347. 21071052 10.1016/j.virol.2010.10.015 PMC7173327

[37] Pogany J, Stork J, Li Z, Nagy PD (2008) In vitro assembly of the Tomato bushy stunt virus replicase requires the host Heat shock protein 70. Proc Natl Acad Sci U S A 105: 19956–19961. 19060219 10.1073/pnas.0810851105 PMC2604936

[38] Russo M, Burgyan J, Martelli GP (1994) Molecular biology of tombusviridae. Adv Virus Res 44: 381–428. 7817878 10.1016/s0065-3527(08)60334-6

[39] Panavas T, Hawkins CM, Panaviene Z, Nagy PD (2005) The role of the p33:p33/p92 interaction domain in RNA replication and intracellular localization of p33 and p92 proteins of Cucumber necrosis tombusvirus. Virology 338: 81–95. 15936051 10.1016/j.virol.2005.04.025

[40] Pathak KB, Sasvari Z, Nagy PD (2008) The host Pex19p plays a role in peroxisomal localization of tombusvirus replication proteins. Virology 379: 294–305. 18684480 10.1016/j.virol.2008.06.044

[41] Jonczyk M, Pathak KB, Sharma M, Nagy PD (2007) Exploiting alternative subcellular location for replication: tombusvirus replication switches to the endoplasmic reticulum in the absence of peroxisomes. Virology 362: 320–330. 17292435 10.1016/j.virol.2007.01.004

[42] Xu K, Huang TS, Nagy PD (2012) Authentic in vitro replication of two tombusviruses in isolated mitochondrial and endoplasmic reticulum membranes. J Virol 86: 12779–12794. 22973028 10.1128/JVI.00973-12 PMC3497632

[43] Carman GM, Han GS (2011) Regulation of phospholipid synthesis in the yeast Saccharomyces cerevisiae. Annu Rev Biochem 80: 859–883. 21275641 10.1146/annurev-biochem-060409-092229 PMC3565220

[44] Csaki LS, Reue K (2010) Lipins: multifunctional lipid metabolism proteins. Annu Rev Nutr 30: 257–272. 20645851 10.1146/annurev.nutr.012809.104729 PMC3738581

[45] Loewen CJ (2012) Lipids as conductors in the orchestra of life. F1000 Biol Rep 4: 4. 22312416 10.3410/B4-4 PMC3270589

[46] Reue K, Dwyer JR (2009) Lipin proteins and metabolic homeostasis. J Lipid Res 50 Suppl: S109–114. 18941140 10.1194/jlr.R800052-JLR200 PMC2674718

[47] Reue K, Donkor J (2007) Genetic factors in type 2 diabetes: all in the (lipin) family. Diabetes 56: 2842–2843. 18042760 10.2337/db07-1288

[48] Carman GM, Han GS (2006) Roles of phosphatidate phosphatase enzymes in lipid metabolism. Trends Biochem Sci 31: 694–699. 17079146 10.1016/j.tibs.2006.10.003 PMC1769311

[49] Han GS, Siniossoglou S, Carman GM (2007) The cellular functions of the yeast lipin homolog PAH1p are dependent on its phosphatidate phosphatase activity. J Biol Chem 282: 37026–37035. 17971454 10.1074/jbc.M705777200

[50] Choi HS, Su WM, Han GS, Plote D, Xu Z, et al (2012) Pho85p-pho80p phosphorylation of yeast pah1p phosphatidate phosphatase regulates its activity, location, abundance, and function in lipid metabolism. J Biol Chem 287: 11290–11301. 22334681 10.1074/jbc.M112.346023 PMC3322823

[51] Mietkiewska E, Siloto RM, Dewald J, Shah S, Brindley DN, et al (2011) Lipins from plants are phosphatidate phosphatases that restore lipid synthesis in a pah1Delta mutant strain of Saccharomyces cerevisiae. FEBS J 278: 764–775. 21205207 10.1111/j.1742-4658.2010.07995.x

[52] Chae M, Han GS, Carman GM (2012) The Saccharomyces cerevisiae Actin Patch Protein App1p Is a Phosphatidate Phosphatase Enzyme. J Biol Chem 287: 40186–40196. 23071111 10.1074/jbc.M112.421776 PMC3504732

[53] Choi HS, Su WM, Morgan JM, Han GS, Xu Z, et al (2011) Phosphorylation of phosphatidate phosphatase regulates its membrane association and physiological functions in Saccharomyces cerevisiae: identification of SER(602), THR(723), AND SER(744) as the sites phosphorylated by CDC28 (CDK1)-encoded cyclin-dependent kinase. J Biol Chem 286: 1486–1498. 21081492 10.1074/jbc.M110.155598 PMC3020757

[54] Han GS, O'Hara L, Siniossoglou S, Carman GM (2008) Characterization of the yeast DGK1-encoded CTP-dependent diacylglycerol kinase. J Biol Chem 283: 20443–20453. 18458076 10.1074/jbc.M802866200 PMC2459283

[55] Han GS, O'Hara L, Carman GM, Siniossoglou S (2008) An unconventional diacylglycerol kinase that regulates phospholipid synthesis and nuclear membrane growth. J Biol Chem 283: 20433–20442. 18458075 10.1074/jbc.M802903200 PMC2459266

[56] Jaag HM, Stork J, Nagy PD (2007) Host transcription factor Rpb11p affects tombusvirus replication and recombination via regulating the accumulation of viral replication proteins. Virology 368: 388–404. 17689583 10.1016/j.virol.2007.07.003

[57] Wang RY, Stork J, Pogany J, Nagy PD (2009) A temperature sensitive mutant of heat shock protein 70 reveals an essential role during the early steps of tombusvirus replication. Virology 394: 28–38. 19748649 10.1016/j.virol.2009.08.003 PMC2776036

[58] Wang RY, Stork J, Nagy PD (2009) A key role for heat shock protein 70 in the localization and insertion of tombusvirus replication proteins to intracellular membranes. J Virol 83: 3276–3287. 19153242 10.1128/JVI.02313-08 PMC2655559

[59] Pogany J, Nagy PD (2008) Authentic replication and recombination of Tomato bushy stunt virus RNA in a cell-free extract from yeast. J Virol 82: 5967–5980. 18417594 10.1128/JVI.02737-07 PMC2395147

[60] Navarro B, Russo M, Pantaleo V, Rubino L (2006) Cytological analysis of Saccharomyces cerevisiae cells supporting cymbidium ringspot virus defective interfering RNA replication. J Gen Virol 87: 705–714. 16476994 10.1099/vir.0.81325-0

[61] Pathak KB, Pogany J, Xu K, White KA, Nagy PD (2012) Defining the Roles of cis-Acting RNA Elements in Tombusvirus Replicase Assembly In Vitro. J Virol 86: 156–171. 22013057 10.1128/JVI.00404-11 PMC3255868

[62] Pathak KB, Pogany J, Nagy PD (2011) Non-template functions of the viral RNA in plant RNA virus replication. Curr Opin Virol 1: 332–338. 22440835 10.1016/j.coviro.2011.09.011

[63] Hwang YT, McCartney AW, Gidda SK, Mullen RT (2008) Localization of the Carnation Italian ringspot virus replication protein p36 to the mitochondrial outer membrane is mediated by an internal targeting signal and the TOM complex. BMC Cell Biol 9: 54. 18811953 10.1186/1471-2121-9-54 PMC2573885

[64] Weber-Lotfi F, Dietrich A, Russo M, Rubino L (2002) Mitochondrial targeting and membrane anchoring of a viral replicase in plant and yeast cells. J Virol 76: 10485–10496. 12239325 10.1128/JVI.76.20.10485-10496.2002 PMC136569

[65] Kovalev N, Pogany J, Nagy PD (2012) A Co-Opted DEAD-Box RNA Helicase Enhances Tombusvirus Plus-Strand Synthesis. PLoS Pathog 8: e1002537. 22359508 10.1371/journal.ppat.1002537 PMC3280988

[66] Pogany J, Panavas T, Serviene E, Nawaz-Ul-Rehman MS, Nagy PD (2010) A high-throughput approach for studying virus replication in yeast. Current Protocols in Microbiology 19: 16J.11.11–16J.11.15. 10.1002/9780471729259.mc16j01s19 21053256

[67] Price BD, Eckerle LD, Ball LA, Johnson KL (2005) Nodamura virus RNA replication in Saccharomyces cerevisiae: heterologous gene expression allows replication-dependent colony formation. J Virol 79: 495–502. 15596842 10.1128/JVI.79.1.495-502.2005 PMC538723

[68] Eastmond PJ, Quettier AL, Kroon JT, Craddock C, Adams N, et al (2011) A phosphatidate phosphatase double mutant provides a new insight into plant membrane lipid homeostasis. Plant Signal Behav 6: 526–527. 21406976 10.4161/psb.6.4.14748 PMC3142382

[69] Eastmond PJ, Quettier AL, Kroon JT, Craddock C, Adams N, et al (2010) Phosphatidic acid phosphohydrolase 1 and 2 regulate phospholipid synthesis at the endoplasmic reticulum in Arabidopsis. Plant Cell 22: 2796–2811. 20699392 10.1105/tpc.109.071423 PMC2947160

[70] Nakamura Y, Koizumi R, Shui G, Shimojima M, Wenk MR, et al (2009) Arabidopsis lipins mediate eukaryotic pathway of lipid metabolism and cope critically with phosphate starvation. Proc Natl Acad Sci U S A 106: 20978–20983. 19923426 10.1073/pnas.0907173106 PMC2791602

[71] Mine A, Okuno T (2012) Composition of plant virus RNA replicase complexes. Curr Opin Virol 2: 669–675. 23083891 10.1016/j.coviro.2012.09.014

[72] Henry SA, Kohlwein SD, Carman GM (2012) Metabolism and regulation of glycerolipids in the yeast Saccharomyces cerevisiae. Genetics 190: 317–349. 22345606 10.1534/genetics.111.130286 PMC3276621

[73] Grangeon R, Jiang J, Laliberte JF (2012) Host endomembrane recruitment for plant RNA virus replication. Curr Opin Virol 2: 683–690. 23123078 10.1016/j.coviro.2012.10.003 PMC7185485

[74] Laliberte JF, Sanfacon H (2010) Cellular remodeling during plant virus infection. Annu Rev Phytopathol 48: 69–91. 20337516 10.1146/annurev-phyto-073009-114239

[75] de Castro IF, Volonte L, Risco C (2013) Virus factories: biogenesis and structural design. Cell Microbiol 15: 24–34. 22978691 10.1111/cmi.12029 PMC7162364

[76] Sharma M, Sasvari Z, Nagy PD (2011) Inhibition of phospholipid biosynthesis decreases the activity of the tombusvirus replicase and alters the subcellular localization of replication proteins. Virology 415: 141–152. 21561636 10.1016/j.virol.2011.04.008 PMC3107895

[77] Harris TE, Finck BN (2011) Dual function lipin proteins and glycerolipid metabolism. Trends Endocrinol Metab 22: 226–233. 21470873 10.1016/j.tem.2011.02.006 PMC3118913

[78] van Harmelen V, Ryden M, Sjolin E, Hoffstedt J (2007) A role of lipin in human obesity and insulin resistance: relation to adipocyte glucose transport and GLUT4 expression. J Lipid Res 48: 201–206. 17035674 10.1194/jlr.M600272-JLR200

[79] Yao-Borengasser A, Rasouli N, Varma V, Miles LM, Phanavanh B, et al (2006) Lipin expression is attenuated in adipose tissue of insulin-resistant human subjects and increases with peroxisome proliferator-activated receptor gamma activation. Diabetes 55: 2811–2818. 17003347 10.2337/db05-1688

[80] Sasvari Z, Izotova L, Kinzy TG, Nagy PD (2011) Synergistic Roles of Eukaryotic Translation Elongation Factors 1Bgamma and 1A in Stimulation of Tombusvirus Minus-Strand Synthesis. PLoS Pathog 7: e1002438. 22194687 10.1371/journal.ppat.1002438 PMC3240602

[81] Li Z, Barajas D, Panavas T, Herbst DA, Nagy PD (2008) Cdc34p ubiquitin-conjugating enzyme is a component of the tombusvirus replicase complex and ubiquitinates p33 replication protein. J Virol 82: 6911–6926. 18463149 10.1128/JVI.00702-08 PMC2446948

[82] Shah Nawaz-Ul-Rehman M, Reddisiva Prasanth K, Baker J, Nagy PD (2013) Yeast screens for host factors in positive-strand RNA virus replication based on a library of temperature-sensitive mutants. Methods 59: 207–216. 23147170 10.1016/j.ymeth.2012.11.001

[83] Pathak KB, Jiang Z, Ochanine V, Sharma M, Pogany J, et al (2013) Characterization of dominant-negative and temperature-sensitive mutants of tombusvirus replication proteins affecting replicase assembly. Virology 437: 48–61. 23332599 10.1016/j.virol.2012.12.009

[84] Panavas T, Nagy PD (2003) Yeast as a model host to study replication and recombination of defective interfering RNA of Tomato bushy stunt virus. Virology 314: 315–325. 14517084 10.1016/s0042-6822(03)00436-7

[85] Barajas D, Li Z, Nagy PD (2009) The Nedd4-type Rsp5p ubiquitin ligase inhibits tombusvirus replication by regulating degradation of the p92 replication protein and decreasing the activity of the tombusvirus replicase. J Virol 83: 11751–11764. 19759160 10.1128/JVI.00789-09 PMC2772669

[86] Rieder SE, Emr SD (2001) Isolation of subcellular fractions from the yeast Saccharomyces cerevisiae. Curr Protoc Cell Biol Chapter 3: Unit 3 8. 10.1002/0471143030.cb0308s08 18228360

[87] Panavas T, Serviene E, Pogany J, Nagy PD (2008) Genome-wide screens for identification of host factors in viral replication. Methods Mol Biol 451: 615–624. 18370284 10.1007/978-1-59745-102-4_41

[88] Cheng CP, Jaag HM, Jonczyk M, Serviene E, Nagy PD (2007) Expression of the Arabidopsis Xrn4p 5′-3′ exoribonuclease facilitates degradation of tombusvirus RNA and promotes rapid emergence of viral variants in plants. Virology 368: 238–248. 17688902 10.1016/j.virol.2007.07.001

