# Supplementary material for: Inactivation of the Host Lipin Gene Accelerates RNA Virus Replication through Viral Exploitation of the Expanded Endoplasmic Reticulum Membrane
Source: PLoS Pathog. 2014 Feb 20;10(2):e1003944. doi: 10.1371/journal.ppat.1003944 (PMC3930575; doi:10.1371/journal.ppat.1003944)
Supplement: Table S1 — Viruses used in this study. (DOC) [file ppat.1003944.s005.doc]

**Table S1. Viruses used in this study.**

Name Genus replication characteristic

Tomato bushy stunt virus (TBSV) tombusvirus peroxisomal membrane

Cucumber necrosis virus (CNV) tombusvirus peroxisomal membrane

Carnation Italian ringspot virus (CIRV) tombusvirus mitochondrial membrane

Nodamura virus (NoV) alphanodavirus mitochondrial membrane

Red clover necrotic mosaic virus (RCNMV) dianthovirus ER membrane
